# Radiological Mimickers of COVID-19 Pneumonia: A Pictorial Review

**Published:** 2020-11

**Authors:** Mehrdad Bakhshayeshkaram, Sara Haseli, Pooya Iranpour

**Affiliations:** 1 Chronic Respiratory Diseases Research Center, National Research Institute of Tuberculosis and Lung Diseases (NRITLD), Shahid Beheshti University of Medical Sciences, Tehran, Iran,; 2 Medical Imaging Research Center, Department of Radiology, Shiraz University of Medical Sciences, Shiraz, Iran

**Keywords:** COVID-19, Coronavirus, Tomography, X-ray computed tomography

## Abstract

Computed tomography (CT) scan plays an important role in the early diagnosis of coronavirus disease (COVID-19) pneumonia. In resource-limited regions with limited availability of polymerase chain reaction (PCR) kits, CT findings, together with appropriate clinical parameters, are used to establish an accurate diagnosis. However, since the radiological findings are non-specific, the CT features may overlap with the findings of several other categories of pulmonary diseases. Diagnosis based on radiological features can be especially challenging in the presence of a comorbid lung disease. This study aimed to describe the radiological findings of a wide spectrum of lung pathologies, with emphasis on their similarities with the common presentations of COVID-19 pneumonia.

## INTRODUCTION

Since the emergence of a novel coronavirus disease, called coronavirus disease-2019 (COVID-19) in Wuhan, China in December 2019, increasing numbers of patients have been diagnosed with this infection worldwide. The World Health Organization (WHO) declared this infection as a global pandemic. At present, the gold standard for detecting severe acute respiratory syndrome coronavirus-2 (SARS-CoV-2) is reverse transcriptase-polymerase chain reaction (RT-PCR) assay. Nevertheless, imaging, especially computed tomography (CT) scan, plays an essential role in the diagnosis and monitoring of patients with suspected SARS-CoV-2 infection.

There are several guidelines for optimal reporting of radiological findings in cases of COVID-19 pneumonia (1).

**Figure 1.**
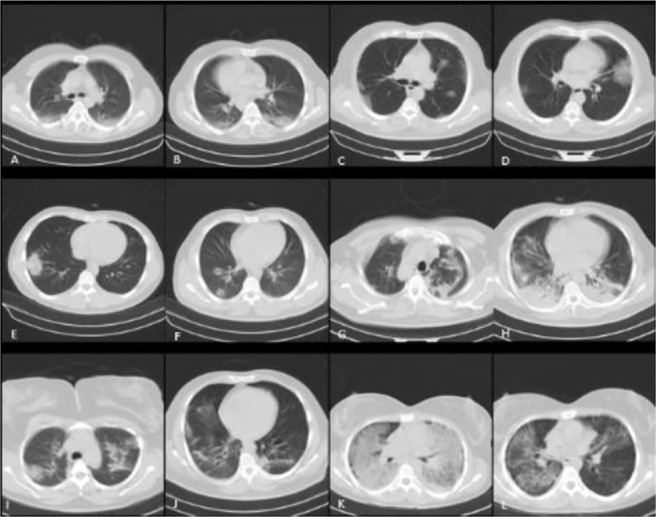
Various appearances of SARS-Cov-2 in CT scan of patients with positive PCR tests and respiratory symptoms: A–B: Typical peripherally located GGO, C–D: GGO in both peri-bronchovascular and peripheral locations. E: isolated consolidation without air-bronchogram sign. F: nodular appearance of GGO in subpleural part of right lower lobe. G: irregular bands affecting apex with consolidation suggestive for sub-acute phase of infection. H: Bi-basilar consolidations resembling aspiration pneumonia. I–J: consolidations with an irregular border and sub-pleural bands. K: diffuse GGO with air-bronchogram and bilateral pleural effusion, this pattern is atypical for SARS-Cov-2. L: GGO with halo sign; Subpleural sparing is also evident as subpleural lucency.

The main imaging findings of SARS-CoV-2 infection, which are detectable on CT scan, include multifocal, multilobar, and peripherally dominant ground-glass opacities (GGO), especially affecting the posterior and basal segments (1). As the number of COVID-19 cases has increased around the world, other non-specific and atypical patterns of SARS -CoV-2 infection have been highlighted in CT scans, including isolated GGO or consolidation, lobar distribution, and nodular patterns. Moreover, several radiological signs, including the halo sign, reversed halo sign, and crazy-paving pattern, have been reported (1). Some of the most important CT manifestations of COVID-19 pneumonia in a series of PCR-confirmed cases are presented in Figure 1.

Table 1 summarizes the most important radiological findings in different cases of COVID-19 pneumonia, according to the Fleischner Society criteria (2). In the context of COVID-19 outbreak, it is possible to make a definite diagnosis by detecting radiological changes and evaluating the clinical signs and symptoms of patients. However, in the presence of a comorbid lung disease, diagnosis based on radiological features can be challenging, as many imaging findings are non-specific and can be detected in diverse groups of lung diseases. Therefore, radiologists are required to be familiar with other conditions, mimicking SARS-CoV-2 infection on CT scans.

**Table 1.** Definition of the CT findings in COVID-19 pneumonia

**Radiologic finding**	**Description**
Ground-glass opacity	Hazy increased lung opacity with preserved bronchovascular margins
Consolidation	Homogeneous increase in pulmonary parenchymal attenuation with obscured margins of vessels and airway walls
Crazy-paving pattern	Thickening of interlobular septa superimposed on a background of ground-glass density.
Interlobular septa	Thin linear opacities between lobules
Halo Sign	Ground-glass opacity surrounding a nodule or mass
Reversed Halo sign	Focal round area of ground-glass density surrounded by a rim of consolidation

This study aimed to discuss and illustrate the radiological findings of a wide spectrum of lung diseases, with emphasis on their similarities with the common presentations of COVID-19 pneumonia.

### Teaching points:

- Radiological findings of COVID-19 pneumonia are non-specific.
- The dominant pattern of COVID-19 infection on CT scan is peripheral GGO with a posterobasal segmental distribution.
- Several categories of diseases, including alveolar, interstitial, and vascular pathologies, may mimic the CT findings of COVID-19 pneumonia.

### Artifacts/physiological mimickers:

**Figure 2.**
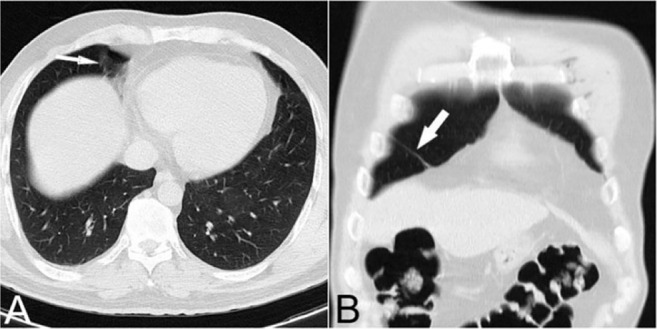
A) Coronal reconstructed image demonstrates a fibrotic band in anterobasal segment of RLL (arrow). B) Corresponding axial image shows artifactual zone of ground-glass density caused by volume averaging of the fibrotic band with adjacent normal lung parenchyma.

While GGOs are typical manifestations of COVID-19 pneumonia, some conditions may stimulate GGO. Inadequate inspiration and motion artifacts, either caused by respiration during scanning or cardiac pulsation, may artifactually produce a GGO appearance (3). Fibrotic bands or subsegmental atelectasis are common findings of chest CT scans, particularly in the lower lobes. They may artifactually present as patches of GGO in one plane, which is due to the partial volume averaging artifact, caused by the presence of materials of different density in the same voxel (Figure 2) (4).

Moreover, an amorphous increase in the density of posterolateral segments of both lower lobes in the subpleural area is a common finding, especially in supine images (5). The reversibility of this physiological increase in attenuation can be easily elucidated via prone scanning (5). However, caution must be taken not to misdiagnose the physiological increase of lung density on the chest CT scans with increased ground glass attenuation in neonates and young children, as the former is usually associated with the underexpansion of an immature lung and the small size of alveoli, resulting in lower air pressure (5).

### Trauma:

**Figure 3.**
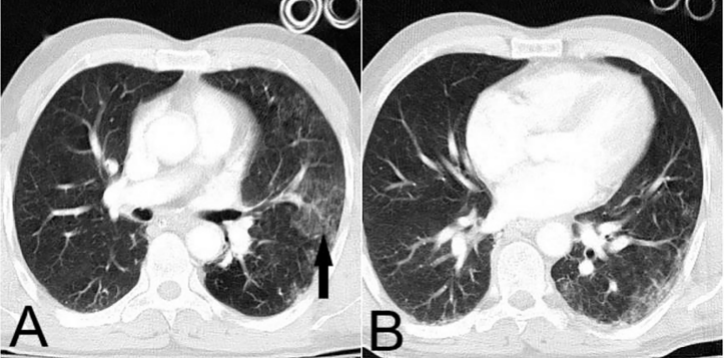
A,B) Peripheral subpleural GGOs in the left lung, represent post-traumatic contusions.

Pulmonary contusion, as the most common injury in blunt chest traumas, is a result of alveolar hemorrhage, but with a preserved alveolar wall. It typically presents as patchy airspace opacities at the site of injury or as a contrecoup contusion on the opposite side (Figure 3) (6). This type of injury has a non-specific appearance, ranging from nodular opacities to larger confluent zones of peripheral consolidation (7). Although it resembles COVID-19 infection in terms of appearance, appropriate clinical history-taking, together with rapid resolution of uncomplicated contusions in the follow-up (7), is helpful in establishing an accurate diagnosis.

### Alveolar lung disease: Pneumonia

#### Viral pneumonia:

**Figure 4.**
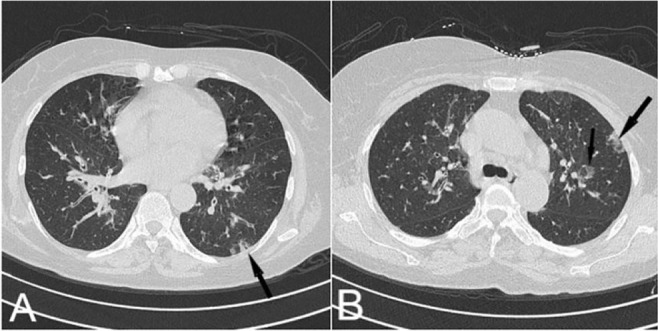
A,B)Non-contrast axial chest CT scan of 42-year-old woman, patchy Peri-bronchovascular GGO and thickening (arrows) is seen during the epidemic of H1N1, compatible with influenza pneumonia.

Viruses are the most common causes of respiratory infections. CT findings are variable and dependent on the pathophysiology of infection, the host’s immune response, and the presence of a superimposed bacterial infection. Multifocal GGOs, consolidations, bronchial and bronchiolar wall thickening, the tree-in-bud sign, and interlobular septal thickening have all been associated with respiratory infections (Figure 4) (8, 9). Although differentiating viral pneumonia from other infections, solely based on CT findings, is challenging and sometimes impossible, it is important to consider viral pneumonia in case of a rapidly progressive infection. Generally, CT scan is performed to assess the extent of the disease and to evaluate the patient’s response to treatment (9).

#### Non-viral pneumonia:

Bacteria are an important cause of community-acquired pneumonia (CAP). The four distinct radiological findings of CAP include lobar consolidations, bronchoalveolar patches, GGO, and rarely random nodules (10). When there is a typical presentation of lobar pneumonia, *Streptococcus pneumoniae* or *Klebsiella pneumoniae* is the most common pathogen. Nevertheless, *Mycoplasma*, *Chlamydia trachomatis*, and *Haemophilus influenzae* may present as peribronchial consolidations (10). Also, *Mycoplasma pneumonia* and *Pneumocystis jirovecii* may predominantly present as GGO patterns (10) and overlap with the radiological manifestations of COVID-19 pneumonia.

#### Non-infectious causes:

**Figure 5.**
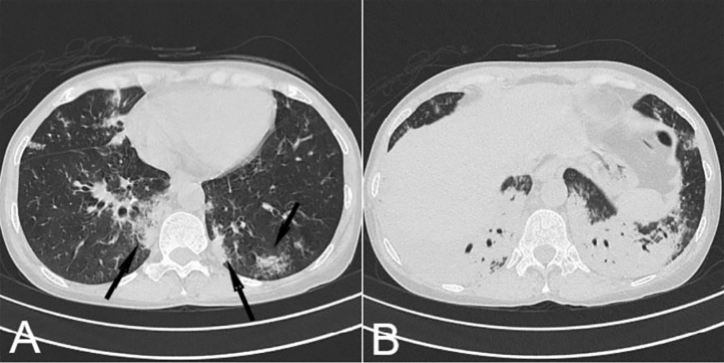
A,B)Bilateral subpleural basal consolidations in a 55-year-old male with a history of CVA and difficulty in swallowing, in favor of aspiration pneumonia.

Aspiration pneumonia, resulting from aspiration of different materials, causes various pulmonary complications, such as lobar and segmental pneumonia, bronchopneumonia, lung abscess, and empyema (11). Considering the type of lung pathology, radiological manifestations, such as ground-glass attenuation, centrilobular branching structures, centrilobular nodules, and thickening of bronchovascular bundles, may vary (Figure 5) (12).

#### Pulmonary edema:

**Figure 6.**
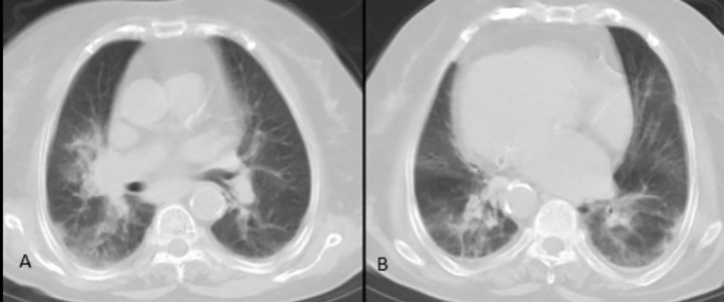
Non-contrast axial chest CT scan of 78-year-old woman Known case of ischemic heart disease showed cardiomegaly, coronary artery calcification, bilateral minimal pleural effusion and para-hilar ground-glass opacities that are typical for cardiopulmonary edema, subsequent echocardiography demonstrated diastolic dysfunction and EF: 35%.

Pulmonary edema is typically classified into two subgroups, based on the underlying etiology. One type of pulmonary edema is hydrostatic pulmonary edema, a characteristic presentation of heart failure, where an increased intravascular hydrostatic pressure results in the extravasation of fluid into the interstitial and alveolar spaces. The most common radiological manifestation of this condition is GGO and consolidation with a central parahilar distribution. The central predominance of radiologic changes is opposed to the more peripheral distribution of COVID-19 pneumonia (Figure 6). This condition is most often seen in ARDS patients and can be due to sepsis, severe trauma, burn, drug overdose, and toxicity (13). CT scan usually reveals inhomogeneous/patchy bilateral GGOs and consolidations with a more gravity-dependent distribution (13).

#### Pulmonary alveolar proteinosis (PAP):

**Figure 7.**
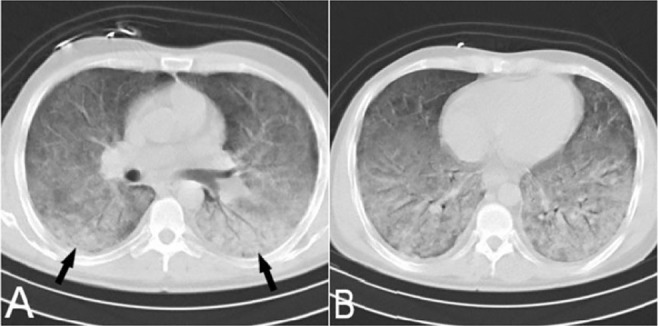
A,B)Non-contrast axial chest CT scan of 36-year-old man, a case of HIV who developed dry cough and fever showed bilateral GGO more dominant in para-hilar locations with no sign of effusion and no lymphadenopathies. PCR result for Bronchoalveolar lavage fluid (BALF) was positive for Pneumocystis pneumonia.

PAP is characterized by the accumulation of surfactant in alveoli. Specific genetic mutations, toxic fume inhalation, and hematological or autoimmune disorders may result in PAP. GGOs, consolidations, and septal reticulations, particularly in the presence of a crazy-paving pattern are highly suggestive of this condition (although non-specific) and warrant specific assessment of the bronchoalveolar lavage fluid (Figure 7) (14). Despite a commonly non-specific distribution, lower zone predominance may be seen in up to 22% of cases (14).

#### Chronic interstitial lung disease (ILD):

**Figure 8.**
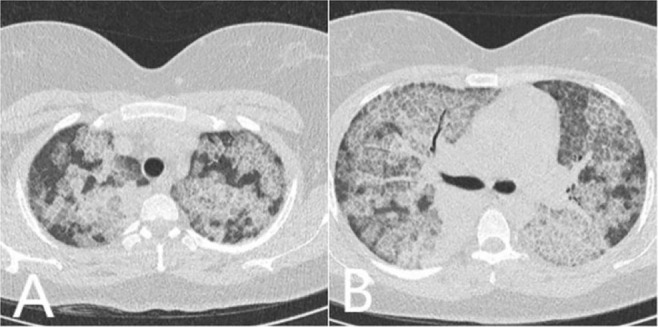
Non-contrast axial chest CT scan of a 34-year-old woman presented with progressive dyspnea and cough for three months. CT demonstrates zones of ground-glass density with interlobular septal thickening, representing typical “crazy-paving” appearance. Bronchoalveolar lavage (BAL) was positive for the accumulation of periodic acid-Schiff (PAS)-positive lipoproteinaceous material.

**Figure 9.**
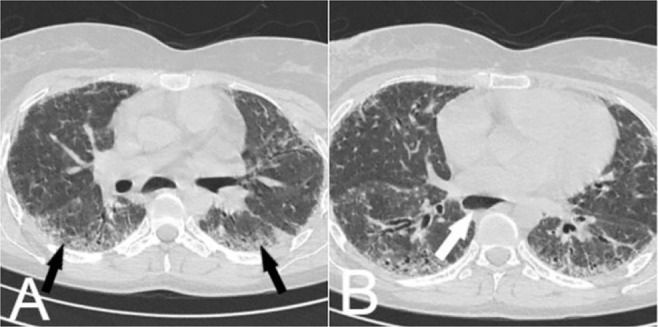
A)Non-contrast axial chest CT scan of 54-year-old woman known case of scleroderma showed sub-pleural GGO, reticulation, bronchiectasis and dilated esophagus (white arrow in B) that are typical for NSIP.

**Figure 10.**
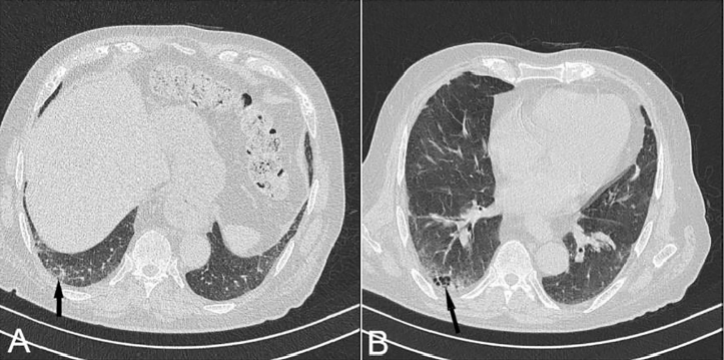
DIP: Non-contrast axial chest CT scan of 54-year-old man, smoker with progressive dyspnea and dry cough showed subpleural patches of GGO.

**Figure 11.**
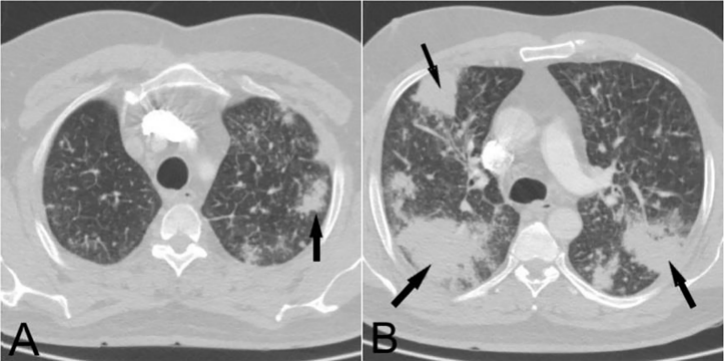
Axial CT scan of a 50-year-old male with a history of cough, dyspnea, and fever for 2months, with no response to antibiotic therapy. Bilateral subpleural consolidations in both lungs are suggestive for COP. Steroid was administered with a significant response to treatment.

ILD is a major contributor to morbidity and mortality, associated with pulmonary diseases. They are ideally evaluated, using high-resolution computed tomography (HRCT) scan. Although imaging findings can be specific in some subgroups, some common radiological findings may be also found (Figures 8–11). The major subtypes of ILD include usual interstitial pneumonia (UIP), hypersensitivity pneumonitis, non-specific interstitial pneumonitis (NSIP), acute interstitial pneumonitis (AIP), desquamative interstitial pneumonitis (DIP), respiratory bronchiolitis-associated ILD (RB-ILD), lymphoid interstitial pneumonia (LIP), and cryptogenic organizing pneumonia (COP) (15). It seems that the peripheral and basilar distribution of parenchymal involvement in UIP and NSIP, associated with interlobular septal thickening and GGO, may mimic the features of COVID-19.

Honeycombing and tractional bronchiectasis, as uncommon features of COVID-19 (at least in the acute phase), may be helpful in differentiation of this infection. COP can be considered as a differential diagnosis when detecting bilateral patchy consolidations with a peripheral distribution (16). This condition also clinically presents with fever, dyspnea, cough, and malaise, similar to COVID-19 pneumonia, and there is no significant response to common treatment protocols.

### Vascular pathologies:

#### Vasculitis:

Pulmonary vasculitides, as a heterogeneous group of diseases, are inflammatory disorders, affecting pulmonary blood vessels of different sizes. Many patients present with multiple organ involvement, and an isolated pulmonary presentation has been rarely reported (17). The imaging findings of pulmonary vasculitis are non-specific and encompass a wide range of radiological manifestations. Diffuse or patchy GGOs (with or without consolidations), cavitary or non-cavitary nodules, centrilobular nodules, and tree-in-bud opacities have all been reported in the literature (17, 18).

**Figure 12.**
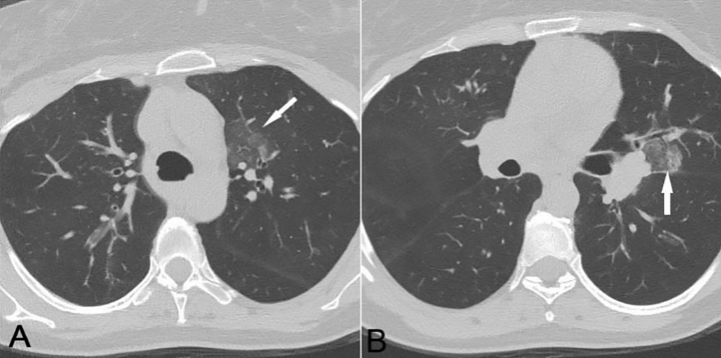
Axial chest CT images of a known case of Churg-Strauss demonstrate patchy transient nonsegmental opacities with no zonal predilection. Air way involvement such as bronchial wall thickening and bronchiectasis may also be detectable.

**Figure 13.**
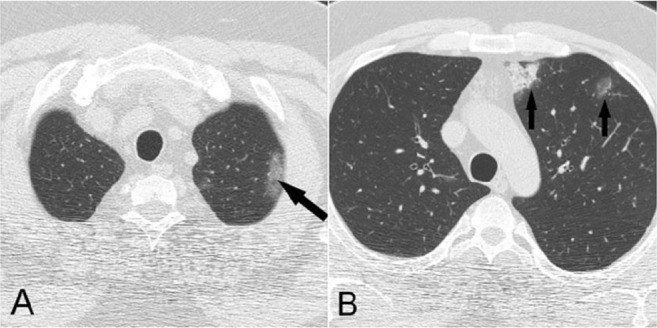
Patches of GGO in a known case of Granulomatosis with polyangiitis. These changes together with nodules, consolidations, and cavitation are the most common abnormalities of ANCA-associated granulomatous vasculitis.

**Figure 14.**
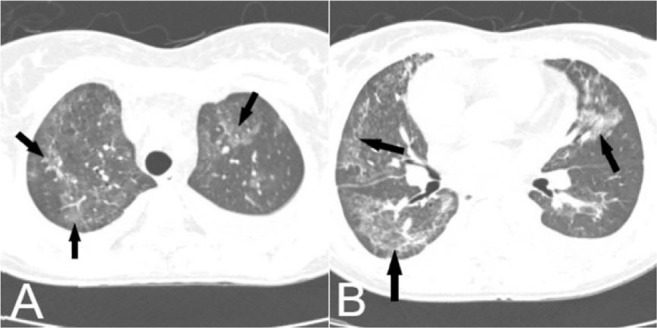
SLE has different lung manifestations such as acute lupus pneumonitis, interstitial lung involvement, alveolar hemorrhage, pulmonary arterial and venous thrombosis. Axial CT images show bilateral zones of GGO and consolidation with septal and interstitial thickening.

Polyarteritis nodosa (PAN), granulomatosis with polyangiitis, Churg-Strauss syndrome, and lupus are several examples of pulmonary vasculitides with pulmonary manifestations (Figures 12–14). Many of these conditions may present with alveolar hemorrhage, which radiologically appears as GGO zones. Evidence shows that a recent hemorrhage can ultimately lead to fibrotic changes (18). Moreover, multifocal, fluctuant, patchy, and nonsegmental consolidation, with no specific zonal predilection, is a common finding of Churg-Strauss syndrome (17, 18). Airway involvement, including bronchial wall thickening, bronchiectasis, and pleural effusion, has been also reported in about 50% of these patients, which is much more common than the rate reported in cases of COVID-19 pneumonia (17).

#### Pulmonary emboli and pulmonary infarction:

Peripherally located ground-glass attenuation, commonly followed by segmental consolidation, is a feature of acute pulmonary emboli, accompanied by pulmonary infarction (18, 19). The emergence of a triangular opacity with a broad base and a linear band, extending from the apex toward the hilum, has been reported as a radiological finding of pulmonary infarction. Moreover, a fan-shaped GGO can be a premonitory sign of this condition (19).

**Figure 15.**
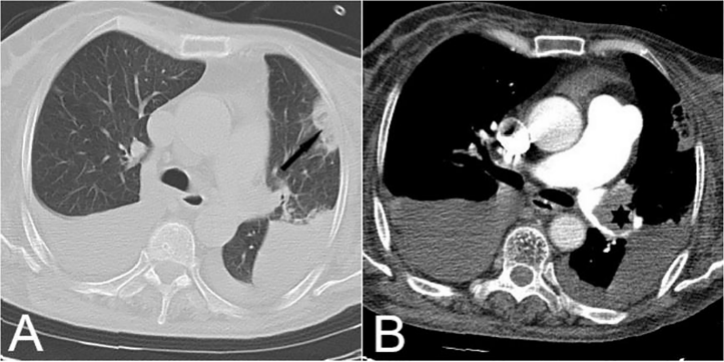
A) Axial lung window CT image demonstrated a patch of subpleural ground-glass change with a rim of consolidation, compatible with the reversed-halo sign. B) Axial mediastinal window, reveals a large filling defect (asterisk) in the left pulmonary artery with secondary pulmonary infarction.

In some cases of pulmonary infarction, the focal area of GGO is surrounded by a complete or incomplete rim of consolidation, giving rise to the reversed halo sign (atoll sign) (Figure 15) (20). Pulmonary infarction was once recognized as a specific sign of COP, but later, it was identified in many other conditions, including pulmonary infections (20). This condition has been also reported in some cases of COVID-19 pneumonia. Nonetheless, the filling defect in the vessel supplying the abnormal zone must be identified for an accurate diagnosis.

**Figure 16.**
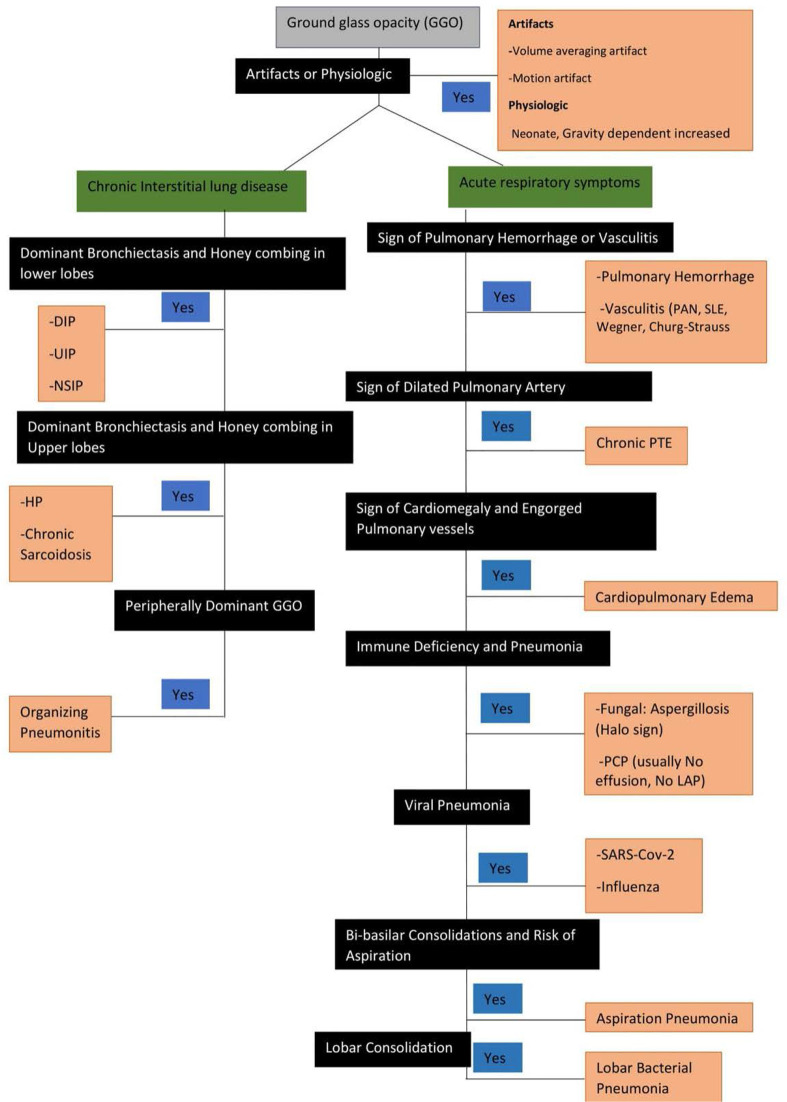
An example of a flowchart for the approach to GGO in chest CT scan.

Generally, the main radiological finding of chronic pulmonary thromboembolism is mosaic attenuation, caused by decreased zonal perfusion and vascular constriction. A normally perfused lung may appear as hyperdense, relative to the oligemic zone, and may be misdiagnosed as a pathological GGO. This condition can be detected by observing the scarcity of vascular density in the low attenuation area and uniform attenuation changes in inspiratory and expiratory CT studies (18). Figure 16 demonstrates an example of approach to GGO in chest CT scan.

## CONCLUSION

COVID-19 pneumonia is typically characterized by multifocal/multilobar zones of GGO, mostly in subpleural areas. The diagnosis of this condition is straightforward in the setting of coronavirus pandemic. However, radiologists should consider other possible differential diagnoses, especially when the clinical scenario is not in favor of viral pneumonia, the patient’s RT-PCR result is negative, or there is an underlying disease.

## References

[1] Simpson S Kay FU Abbara S Bhalla S Chung JH Chung M Radiological Society of North America Expert Consensus Statement on Reporting Chest CT Findings Related to COVID-19. Endorsed by the Society of Thoracic Radiology, the American College of Radiology, and RSNA. Radiology: Cardiothoracic Imaging 2020; 2 (2): e200152. 10.1148/ryct.2020200152 PMC7233447 33778571

[2] Hansell DM Bankier AA MacMahon H McLoud TC Müller NL Remy J. Fleischner Society: glossary of terms for thoracic imaging. Radiology 2008; 246 (3): 697– 722. 18195376 10.1148/radiol.2462070712

[3] Lynch DA. Ground glass attenuation on CT in patients with idiopathic pulmonary fibrosis. Chest 1996; 110 (2): 312– 3. 8697823 10.1378/chest.110.2.312

[4] Lynch DA Newell JD Lee JS Imaging of diffuse lung disease. PMPH-USA; 2000.

[5] Hansell DM. Thin-section CT of the lungs: the Hinterland of normal. Radiology 2010; 256 (3): 695– 711. 20720066 10.1148/radiol.10092307

[6] Kaewlai R Avery LL Asrani AV Novelline RA. Multidetector CT of blunt thoracic trauma. Radiographics 2008; 28 (6): 1555– 70. 18936021 10.1148/rg.286085510

[7] Thoongsuwan N Kanne JP Stern EJ. Spectrum of blunt chest injuries. J Thorac Imaging 2005; 20 (2): 89– 97. 15818207 10.1097/01.rti.0000148210.89718.f5

[8] Koo HJ Lim S Choe J Choi SH Sung H Do KH. Radiographic and CT Features of Viral Pneumonia. Radiographics 2018; 38 (3): 719– 739. 29757717 10.1148/rg.2018170048

[9] Franquet T. Imaging of pulmonary viral pneumonia. Radiology 2011; 260 (1): 18– 39. 21697307 10.1148/radiol.11092149

[10] Nambu A Ozawa K Kobayashi N Tago M. Imaging of community-acquired pneumonia: Roles of imaging examinations, imaging diagnosis of specific pathogens and discrimination from noninfectious diseases. World J Radiol 2014; 6 (10): 779– 93. 25349662 10.4329/wjr.v6.i10.779 PMC4209424

[11] Franquet T Giménez A Rosón N Torrubia S Sabaté JM Pérez C. Aspiration diseases: findings, pitfalls, and differential diagnosis. Radiographics 2000; 20 (3): 673– 85. 10835120 10.1148/radiographics.20.3.g00ma01673

[12] Komiya K Ishii H Umeki K Kawamura T Okada F Okabe E Computed tomography findings of aspiration pneumonia in 53 patients. Geriatr Gerontol Int 2013; 13 (3): 580– 5. 22994842 10.1111/j.1447-0594.2012.00940.x

[13] Miller WT Jr Shah RM. Isolated diffuse ground-glass opacity in thoracic CT: causes and clinical presentations. AJR Am J Roentgenol 2005; 184 (2): 613– 22. 15671387 10.2214/ajr.184.2.01840613

[14] Borie R Danel C Debray MP Taille C Dombret MC Aubier M Pulmonary alveolar proteinosis. Eur Respir Rev 2011; 20 (120): 98– 107. 21632797 10.1183/09059180.00001311 PMC9487789

[15] Bartholmai BJ Raghunath S Karwoski RA Moua T Rajagopalan S Maldonado F Quantitative computed tomography imaging of interstitial lung diseases. J Thorac Imaging 2013; 28 (5): 298– 307. 23966094 10.1097/RTI.0b013e3182a21969 PMC3850512

[16] Nishino M Itoh H Hatabu H. A practical approach to high-resolution CT of diffuse lung disease. Eur J Radiol 2014; 83 (1): 6– 19. 23410907 10.1016/j.ejrad.2012.12.028 PMC3745551

[17] Chung MP Yi CA Lee HY Han J Lee KS. Imaging of pulmonary vasculitis. Radiology 2010; 255 (2): 322– 41. 20413748 10.1148/radiol.10090105

[18] Engelke C Schaefer-Prokop C Schirg E Freihorst J Grubnic S Prokop M. High-resolution CT and CT angiography of peripheral pulmonary vascular disorders. Radiographics. 2002 Jul-Aug; 22 (4): 739– 64. 12110707 10.1148/radiographics.22.4.g02jl01739

[19] Shinohara T Naruse K Hamada N Yamasaki T Hatakeyama N Ogushi F. Fan-shaped ground-glass opacity (GGO) as a premonitory sign of pulmonary infarction: a case report. J Thorac Dis 2018; 10 (1): E55– E58. 29600105 10.21037/jtd.2017.12.64 PMC5863182

[20] Nattusamy L Madan K Khilnani GC Guleria R. Pulmonary infarction in acute pulmonary embolism: reversed halo sign. BMJ Case Rep 2014; 2014: bcr2014205181. 10.1136/bcr-2014-205181 PMC4069759 24957589

